# COMPARATIVE ANALYSIS OF THE EFFECTIVENESS OF CLINICAL DATA COLLECTION THROUGH ONLINE AND PHYSICAL ELECTRONIC QUESTIONNAIRE IN ORTHOPEDIC PATIENTS

**DOI:** 10.1590/1413-785220233105e268380

**Published:** 2023-12-18

**Authors:** ANDRE VITOR KERBER CAVALCANTI LEMOS, LUCAS PLENS DE BRITTO COSTA, EDUARDO SOUZA MACIEL, NACIME SALOMÃO BARBACHAN MANSUR, MOISES COHEN

**Affiliations:** 1 Universidade Federal de Sao Paulo, Centro de Traumatologia do Esporte, Departamento de Ortopedia e Traumatologia, Sao Paulo, SP, Brazil; 2 Hospital Israelita Albert Einstein, Grupo de Ortopedia Pediatrica, Sao Paulo, SP, Brazil

**Keywords:** Sprains and Strains, Ankle, Internet, Data Collection, Entorses e Distensões, Tornozelo, Internet, Coleta de Dados

## Abstract

The collection of clinical data is an essential step for the development of any scientific research. Online digital data collection can optimize this step.

**Objective:**

To compare the response rate and the accuracy of the clinical data collection date through the online and physical digital questionnaire in orthopedic patients.

**Methods:**

Comparative study, level III of evidence, with forty patients who had ankle sprains were evaluated, followed up for a period of 12 weeks with the application of physical and digital Visual Analogue Scale, Foot Function Index and Cumberland Ankle Instability Tool questionnaires, and data were collected about the moment of collection of each questionnaire.

**Results:**

We obtained a response rate of 83.3% in the digital collection group and 60% in the physical collection group (p < 0.05), and the response rate in the digital collection group was higher at all times of collection (3, 6 and 12 weeks). Analysis of the time of collection shows greater variability in the larger physical collection group at all times of the study (2.8 vs 1.5; 4.0 vs 2.4; 8.6 vs 1.5).

**Conclusion:**

Digital data collection is effective for obtaining clinical data in patients with ankle sprains. ***Level of Evidence III, Comparative, Prospective, Longitudinal Study in Parallel Groups.***

## RESUMO

A coleta de dados clínicos é etapa essencial para o desenvolvimento de qualquer pesquisa científica, e a coleta de dados digital online pode otimizá-la.

**Objetivo:**

Comparar o índice de resposta e a precisão da data de coleta de dados clínicos por meio de aplicação de questionário digital online e físico a pacientes ortopédicos.

**Métodos:**

Estudo comparativo realizado com 40 pacientes que apresentaram entorse de tornozelo, acompanhados pelo período de 12 semanas, com aplicação dos questionários escala visual analógica, foot function index e Cumberland ankle instability tool físicos e digitais. Além disso, foram recolhidos dados sobre o momento da coleta dos questionários.

**Resultados:**

Obtivemos índice de resposta de 83,3% no grupo de coleta digital e 60% no grupo de coleta física (p < 0,05), sendo que o índice de resposta no grupo de coleta digital foi maior em todos os momentos de coleta (3, 6 e 12 semanas). A análise do momento da coleta apresenta maior variabilidade no grupo de coleta física em todos os momentos do estudo (2,8 vs 1,5; 4,0 vs 2,4; 8,6 vs 1,5).

**Conclusão:**

A coleta de dados digital é efetiva para a obtenção dos dados clínicos de pacientes que apresentam entorse do tornozelo. **Nível de Evidência III, Estudo Comparativo, Prospectivo, Longitudinal em Grupos Paralelos.**

## INTRODUCTION

The collection of clinical data is an essential step for the development of any scientific research^1^ ^),(^ ^2^. However, the loss of data from clinical follow-up in research is a concern in the literature, occurring in up to 89% of studies, and in around 48% of these studies, data loss greater than 10%^1^ ^)-(^ ^3^ was reported. Recruiting patients to research centers to obtain this data can represent a great difficulty in some situations, especially when collecting frequent or long-term data. ^(^ ^3^ ^).(^ ^4^

The use of information technologies such as the internet can optimize the application of questionnaires, reduce the time to obtain data and reduce the loss of follow-up data. ^(^ ^5^ ^), (^ ^6^ ^), (^ ^7^ ^), (^ ^8^ ^), (^ ^9^ ^), (^ ^10^ ^), (^ ^11^ The use of these questionnaires has already been validated in clinical research^12^ and presents reliable information, ^(^ ^10^ ^),(^ ^13^ ^), (^ ^14^ ^), (^ ^15^ ^), (^ ^16^ and can even be used in orthopedic patients. ^(^ ^17^ ^), (^ ^18^ ^), (^ ^19^ ^), (^ ^20^ ^), (^ ^21^

Ankle sprains are among the most prevalent injuries in the population, ^(^ ^22^ ^),(^ ^23^ account for up to 14% of emergency consultations, and have a high impact on the healthcare system^24^ in addition to progressing to chronic ankle instability in up to 30-40%.^25^ ^),(^ ^26^. Adequate clinical follow-up of these patients is important to assess the possible unfavorable evolution of the condition, ^(^ ^27^ ^),(^ ^28^ although it is common for patients themselves to abandon orthopedic follow-up early, as soon as their pain improves. ^(^ ^26^

The objective of this study is to compare the proportion of responses to the self-administered questionnaires Visual Analogue Scale (VAS), ^(^ ^29^ FFI (Foot Function Index) ^(^ ^30^ and CAIT (Cumberland Ankle Instability Tool) ^(^ ^31^ in two different ways: physically at a medical appointment and applied with a digital online form remotely.

## METHODS

A comparative, prospective, longitudinal study in parallel groups, approved by the Research Ethics Committee of the Universidade Federal de São Paulo (CEP-UNIFESP) and included in Plataforma Brasil under number 1541/2018, following the recommendations of Strengthening the Reporting of Observational Studies in Epidemiology - STROBE. The study was carried out at the Centro de Traumatologia Esportiva of the Departamento de Ortopedia e Traumatologia (DOT-UNIFESP).

Patients with acute ankle ligament sprain/injury (< 15 days) between July and October 2018, with clinical signs of ankle ligament injury, aged between 14 and 65 years, were included. Exclusion criteria were fractures or previous surgeries on the affected limb, associated injuries, difficulty accessing the internet, difficulty understanding the questionnaires, refusal to participate in the study or not agreeing with the consent form, signs of reflex sympathetic dystrophy. Regardless of the group selected for follow-up, all patients followed the same treatment protocol: protection of the limb with immobilization with a semi-rigid ankle brace for a period of 6 weeks, use of analgesic medication as necessary and early rehabilitation.

Patients were instructed on how to use the ankle brace (with socks and lace-up sneakers, nighttime use, and removal only for bathing), relative rest (for heavy physical and work activities) and outpatient follow-ups at 3, 6 and 12 weeks. The patient was allowed partial or total weight bearing with immobilization, as tolerated by the pain, and instructed to begin rehabilitation with physiotherapy, which should be maintained over the 12 weeks.

### Physical/in-person questionnaire group

The first 20 patients included had their data collected through physical questionnaires from the initial assessment to the proposed final follow-up.

This first group responded to questionnaires during outpatient follow-ups scheduled at 3 weeks, 6 weeks and 12 weeks after their initial trauma. At the 3-week follow-up, the VAS and FFI questionnaires were applied, with a tolerance of 1 week (14 to 28 days post-sprain) for data collection. At the 6-week follow-up, the tolerance for data collection was 2 weeks (29 to 56 days post-sprain). At the 12-week follow-up, the tolerance for data collection was set at 3 weeks (63 and 105 days post-sprain). At that moment, in addition to the application of the VAS and FFI, the patient was instructed to answer the CAIT questionnaire. Whenever the patient had an appointment scheduled, attempts were made to contact them by phone and texting to remind them of the appointment.

### Online questionnaire group

The subsequent 20 patients had their data collected through online questionnaires from the initial assessment to the proposed final follow-up. Patients filled out the online questionnaire in the presence of the researcher, during the initial assessment, so that any doubts regarding completion or access could be clarified.

They were informed that they would receive links via cell phone message via the WhatsApp® application or SMS, in addition to an email message, with access to online digital questionnaires on the date to be answered through a cell phone, tablet or computer. When the response to the digital questionnaire was not observed on the set date, patients were contacted via telephone calls or new messages. Patients received a reminder by texting and email on the exact days they completed 3, 6 and 12 weeks, with links to access the questionnaires. Responses were considered valid only when they respected the tolerance periods determined for data collection, similar to the physical data collection group.

Patients selected for the digital questionnaire group responded using an online form created for the study, containing exactly the same questions as the physical questionnaires, with the possibility of answering via smartphone or computers connected to the Internet. In the 3-week and 6-week messages, patients received the following attached link: https://goo.gl/forms/vedkf1SkK982YqF03.

**Figure 1:**
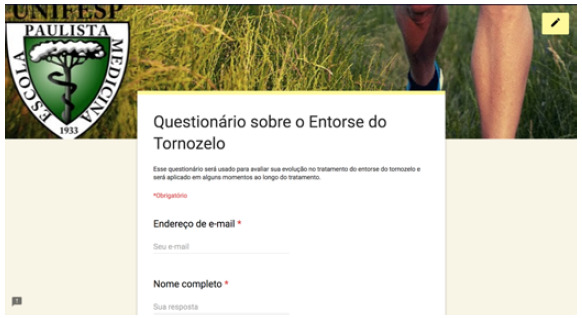
Online questionnaire - identification

**Figure 2:**
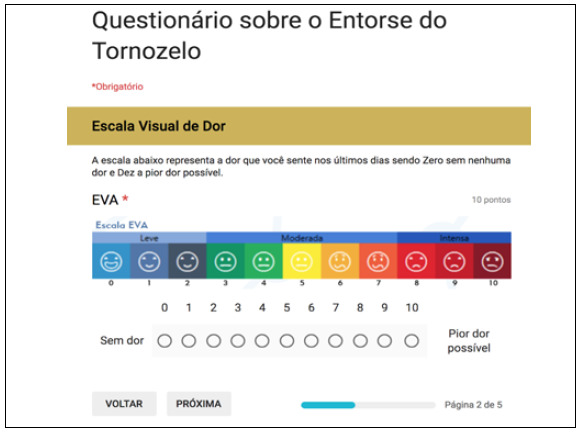
Online questionnaire - VAS

**Figure 3:**
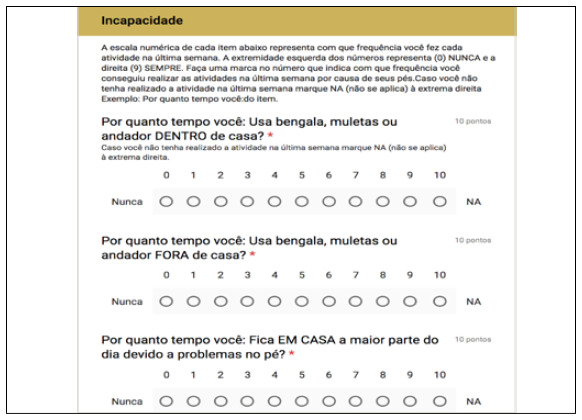
Online questionnaire - FFI (part).

**Figure 4:**
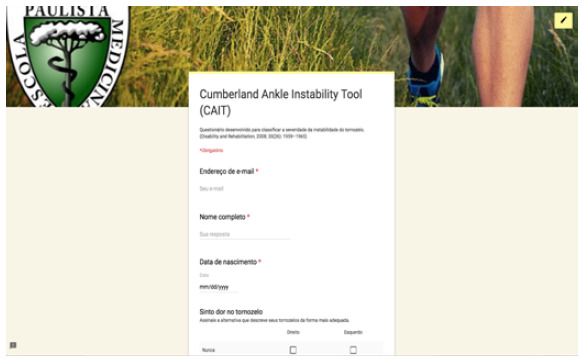
Online questionnaire - CAIT (part).

The questionnaire developed on Google Forms for free is a combination of VAS and FFI (translated into Portuguese), in addition to basic identification data (full name, date of birth and email), partially shown here in Figures 1, 2 and 3. In the 12-week message, in addition to the above-mentioned link, patients received the following link: https://goo.gl/forms/Sia2Iy62wbRF51jx2, which gives access to the questionnaire also developed on Google Forms*,* with the CAIT questions (translated into Portuguese) partly shown here in Figure 4 Statistical analysis was carried out with parametric tests using the programs SPSS V20, Minitab 16 and Excel Office 2010, having established a significance level of 5% (p < 0.05), and adjusted confidence interval (95% CI).

## RESULTS

### Comparison of response rates

In the digital collection group, responses were collected in 50 (83.3%) of the 60 possible questionnaires, while in the physical collection group the response rate was 36 (60%) of the questionnaires (p = 0.005). When segmenting the analysis of the response rate, we noticed that, at the three moments, it was always higher for the Digital Collection group, but statistically significant only in the 6-week collection (80% for digital collection versus 50% for physical collection, p = 0.047) (Table 1).

**Table 1:** Response rates.

Collection days	Digital collection		Physical collection		P-value
	N	%	N	%	
3 weeks	18	90%	13	65%	0.058
6 weeks	16	80%	10	50%	0.047
12 weeks	16	80%	13	65%	0.288
Total	50	83.3%	36	60%	0.005

### Comparison of days for data collection

When evaluating the collection day for each questionnaire, we analyzed the collection days in relation to the proposed ideal day. We observed that the mean collection day is very close to the ideal collection day in both groups. The Mann-Whitney test did not indicate any difference in group means, as observed in Table 2. When analyzing the appropriate patterns, we noticed that these, at all times of collection, are higher in the physical collection group, which means a greater variability of days in relation to the ideal collection day. With this observation, we performed the homoscedasticity analysis. When comparing the variability of collection days between the groups in relation to the ideal day, we observed that there is a difference in the variability of collection days between the groups at 3 weeks (p = 0.003) and also at 12 weeks (p < 0.001) (Table 3).

**Table 2:** Compares groups for “collection days” by moment.

Collection days		Mean	Median	Standard deviation	N	P-value Group
3 weeks	Digital collection	21.5	21	1.5	18	0.663
	Physical collection	22.2	23	2.8	13	
6 weeks	Digital collection	42.3	43	2.4	16	0.669
	Physical collection	41.9	42	4.0	10	
12 weeks	Digital collection	84.7	85	1.5	16	0.387
	Physical collection	83.2	81	8.6	13	

**Table 3:** Compares groups for “collection days” variability

Collection days		Mean	Standard deviation	P-value
3 weeks	Digital collection	21.5	1.54	0.003
	Physical collection	22.2	2.82	
6 weeks	Digital collection	42.3	2.39	0.071
	Physical collection	41.9	4.01	
12 weeks	Digital collection	84.7	1.54	<0.001
	Physical collection	83.2	8.57	

### Patient evolution

No statistically significant differences were found in the VAS, FFI and CAIT measurements between the physical and online collection groups. Regarding the evolution of scores in each group, we concluded that there was a significant reduction in the VAS and a significant progressive increase in the FFI in both groups, in a similar way between them.

## DISCUSSION

Our study compared data collection from online digital questionnaires and physical questionnaires in orthopedic patients. Despite the advantages of using technology to collect clinical data, these tools are little explored in developing countries, ^(^ ^32^ including Brazil. Data collection rates were found to be higher when using online digital questionnaires (83.3%) compared to data collected in physical questionnaires (60%). This finding contradicts studies that compare the application of online and in-person physical questionnaires, ^(^ ^33^ ^),(^ ^34^ which present mean response rates of 33% and 56%, but none of these studies was carried out in the context of medical monitoring, with the physical questionnaire being applied upon follow-up visits. A possible explanation for the advantage of the online digital questionnaire in our study is the fact that it increases the opportunities to respond to the questionnaire, since the patient could answer it at any time, and from anywhere with internet access, in addition to new messages being sent in the absence of responses. On the other hand, the response to the physical questionnaire was necessarily carried out during the follow-up visit.

Many factors can influence response rates to questionnaires administered over the Internet, and we observed that depending on the methodology used, these can be very low. ^(^ ^33^ In our study, we obtained a response rate of 83.3% to the online questionnaire, and the questionnaire was sent to patients who were undergoing orthopedic treatment for a recent injury, and reminder messages were used. Strategies to increase response rates to online questionnaires have already been found to be effective in previous studies^33^ and were used in our study.

In the literature, improvements in obtaining data with online questionnaires had already been observed in studies with geographic obstacles and in remote areas. ^(^ ^9^ ^),(^ ^10^ In our study we noticed that routine problems in large urban centers can also make it difficult to carry out face-to-face interviews.

A high rate of abandonment of conservative treatment for ankle ligament injuries is already known in the literature, ^(^ ^22^ ^),(^ ^28^ and this may be a factor that has influenced the low response rate to the physical questionnaire.

A new finding from our study was the reduction in the variability of collection dates, providing greater precision in dates using the internet. We found a decrease in standard deviation by 45% (2.8 to 1.54) in week 3, by 40% (4.01 to 2.39) in week 6 and by 82% (8.57 to 1.54) in week 12, and this piece of data is still little explored in the literature. A likely explanation for this reduction in the variability of the collection date in the online digital group is the fact that collection can be carried out on any day, including weekends and holidays, while outpatient data collection depends on the flexibility of the schedule of research centers and researchers.

In our study, we did not find any impact on the comparative evaluation of results between groups regarding the variability of the day on which the questionnaires were collected. Probably, the fact that the average collection dates were close to the ideal date minimized possible changes that could appear in the results. The improvement in collection precision may represent a benefit in the quality of the data obtained, but further studies are needed.

As described in the literature, data collection through digital and physical questionnaires does not significantly alter the results of the data obtained. ^(^ ^13^ ^),(^ ^14^ The similarity between the data collected can also be observed when comparing the evolution of VAS and FFI scores between the groups. As previously reported in the literature, ^(^ ^35^ ^),(^ ^36^ we described a high rate of residual symptoms in patients with ligament injuries treated with immobilization, which in our study was observed by the CAIT score (mean 22 in the digital group and 20.33 in the physical group) of the injured ankles after 12 weeks of treatment.

The CFM regulation, through resolution number 2,227/2018, allowed health care to make use of advances in technology, and defined telemedicine as the provision of technology-mediated medical services. ^(^ ^37^ However, many questions and suggestions for changes were sent to the CFM, which revoked this resolution for a more in-depth study of the topic. ^(^ ^38^ With the occurrence of the COVID-19 virus pandemic, this discussion was expanded, and in 2022 the practice of telemedicine was again regulated by the CFM. ^(^ ^39^

Our study’s strong point is the observation of the practical effectiveness of successfully using DRPs to collect data with online questionnaires in orthopedic patients.

Negative points are the lack of data collection regarding the reason for loss of follow-up in both groups, difficulty in analyzing the impact of the variability of collection days on the results of the DRPs, and the lack of randomization for assigning patients to the groups.

A future objective is to create an automated tool for collecting follow-up data on orthopedic patients, which could facilitate data collection by reducing errors in filling out questionnaires and increasing precision at specific moments in the follow-up.

## CONCLUSION

The use of online digital questionnaires is effective for data collection and can be useful for orthopedic patients’ clinical follow-up.

Using the internet not only optimizes information collection but can also increase data accuracy by reducing time of collection variability.
